# Clinical Challenges in Managing Bilateral Acute Iris Transillumination: Insights From a Case Triggered by Fluoroquinolone Therapy

**DOI:** 10.7759/cureus.78442

**Published:** 2025-02-03

**Authors:** Sina Hakami, Youssef Afifi, Majda Rachdi, Xavier Dominguez y Costa, Aurélie Le

**Affiliations:** 1 Department of Ophthalmology, Erasmus Hospital, Brussels, BEL; 2 Department of Ophthalmology, Centre Hospitalier Universitaire (CHU) Saint-Pierre, Brussels, BEL

**Keywords:** bilateral acute depigmentation of iris, bilateral acute iris transillumination, bullous keratopathy, fluoroquinolone therapy, intraocular hypertension, iris depigmentation

## Abstract

Bilateral acute iris transillumination (BAIT) is a rare ocular condition characterized by acute pigment dispersion from the iris pigment epithelium (IPE), resulting in diffuse iris transillumination, atonic mydriatic pupils, and intraocular hypertension. BAIT is often associated with severe photophobia and requires extended medical management. Differentiating BAIT from related conditions such as bilateral acute depigmentation of the iris (BADI) is critical, as BAIT presents with a more severe clinical course and lasting complications. Trigger factors include respiratory infections and fluoroquinolone use, particularly moxifloxacin. This report details a case of BAIT following oral moxifloxacin use. A 69-year-old male patient presented with bilateral ocular redness, pain, and severe photophobia one day after receiving an influenza vaccination and one-week post-moxifloxacin treatment. Examination revealed bullous keratopathy and bilateral iris depigmentation with transillumination. Moreover, Goldmann applanation tonometry showed an elevated intraocular pressure (IOP) exceeding 35 mmHg. Initial management included antiviral, anti-inflammatory, and IOP-lowering agents, which improved symptoms but revealed persistent pigment dispersion, atonic pupils, and recurrent IOP elevation during corticosteroid tapering. Long-term treatment with carbonic anhydrase inhibitors and alpha-adrenergic blockers stabilized IOP, but persistent iris atrophy and pigment dispersion were noted after one year. Therefore, this case underscores the severe and persistent nature of BAIT, emphasizing the importance of distinguishing it from BADI and other conditions with overlapping features. Corticosteroid management requires careful tapering to prevent symptomatic recurrences, and long-term monitoring is crucial to avoid sequelae such as glaucomatous damage. The association with moxifloxacin highlights the need for caution when prescribing fluoroquinolones, especially in at-risk populations, to mitigate potential ocular morbidity.

## Introduction

Bilateral acute iris transillumination (BAIT) is a clinical entity characterized by acute pigment dispersion originating from the iris pigment epithelium (IPE), leading to diffuse iris transillumination. First formally described by Tugal-Tutkun et al. in 2011 [1], BAIT typically presents with acute-onset severe photophobia, conjunctival hyperemia, and pigment dispersion in the anterior chamber. Clinical features include atonic mydriatic pupils unresponsive to light and persistent intraocular hypertension caused by dense pigment deposition in the trabecular meshwork [2]. These complications often require long-term medical management or surgical intervention.

A closely related condition, bilateral acute depigmentation of the iris (BADI), is also characterized by acute pigment dispersion but involves the iris stroma rather than the IPE. Unlike BAIT, BADI does not lead to transillumination defects or pupillary abnormalities and generally follows a milder, self-limiting course with transient intraocular pressure (IOP) elevation [2]. While BAIT is associated with a more severe clinical course and lasting sequelae, the distinction between the two conditions can be challenging due to overlapping features [2].

Both BAIT and BADI have been associated with various potential triggers, including respiratory tract infections [1,3,4], genetic factors [5], and the use of fluoroquinolone antibiotics, particularly moxifloxacin [3,4,6]. The exact etiopathogenesis still remains unclear, and multiple case reports suggest that these conditions affect individuals of various ethnic backgrounds with a predominant involvement of women [2]. We present a case of BAIT in a patient who developed the condition following oral moxifloxacin use.

This report was previously presented as a poster at the 2024 Controversies in Ophthalmology (COPHy) Congress on March 15 and 16, 2024.

## Case presentation

Our case concerns a 69-year-old male patient who was referred to the ophthalmology department for bilateral ocular redness, pain, and photophobia. Symptoms appeared a day after receiving influenzae vaccination and one week after undergoing treatment with oral moxifloxacin, prescribed by his family doctor for a lung infection. Prior to presenting to the emergency ophthalmology department, the patient had no history of ophthalmologic disease or similar attacks.

On slit lamp examination, the patient exhibited bilateral palpebral edema, important chemosis, and diffuse bullous keratopathy, with an IOP estimated to be higher than 35 mmHg on bi-manual palpation since applanation tonometry was unfeasible. Fundus examination was also unachievable. Initial diagnosis leaned towards herpes virus activation: oral acyclovir, oral acetazolamide, and tetracyclin ointment were started. A subsequent check after 24 hours showed no improvement, leading to a prescription of methylprednisolone 64 mg, tobramycine-dexamethasone drops, cyclopentolate drops, and continued oral acyclovir as a precautionary measure.

Photophobia, palpebral edema, and chemosis were greatly resolved under treatment two days later. However, three large keratic bubbles remained on the right eye.

**Figure 1:**
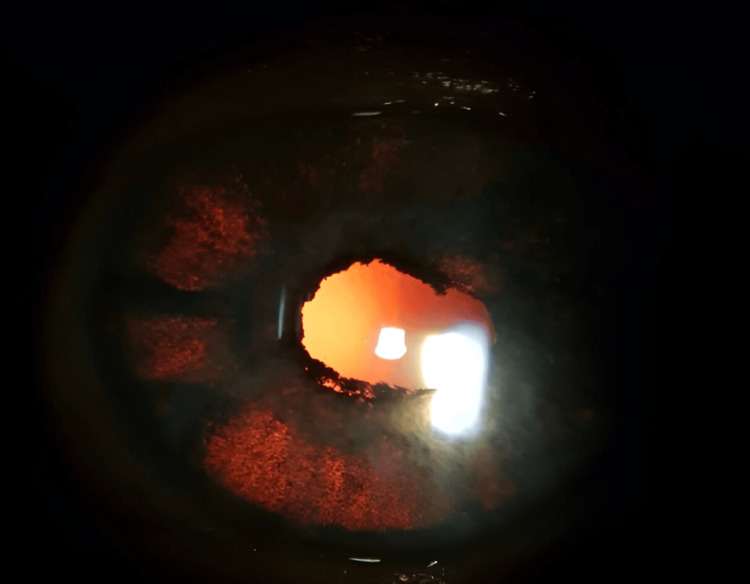
Slit lamp photograph of the right eye showing diffuse iris transillumination defects with large patches of atrophy and posterior synechia

**Figure 2:**
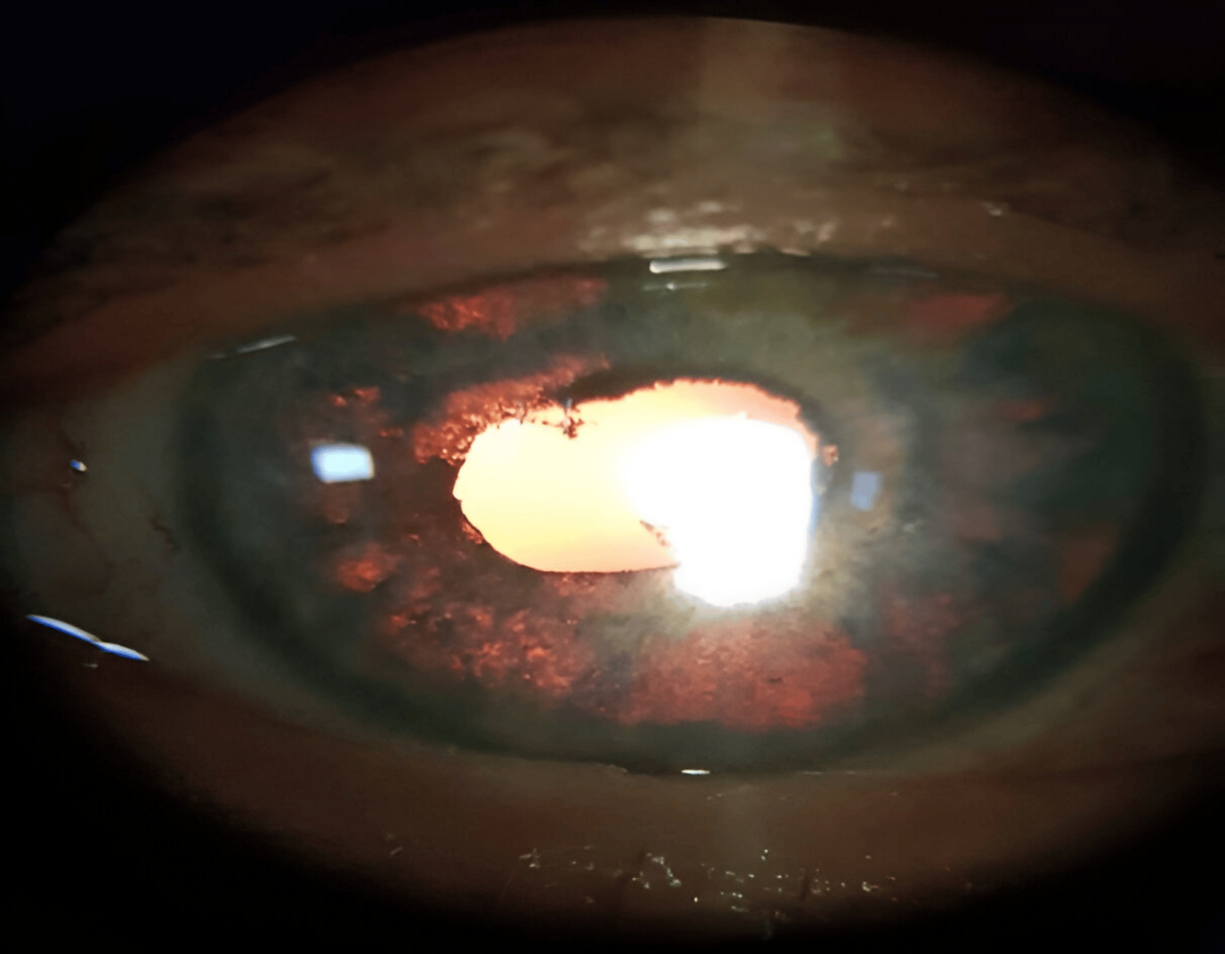
Slit lamp photograph of the left eye showing diffuse iris transillumination defects with large patches of atrophy and posterior synechia

One week later, the examination showed a clear cornea without epithelial blisters, atonic mydriatic pupils with posterior synechiae on both eyes, pigment cells in the anterior chamber, and bilateral diffuse iris depigmentation with transillumination (Figures 1, 2). Visual acuity was measured, using the Snellen chart, at 0.4 in the right eye and 0.8 in the left eye, and applanation tonometry measure showed an IOP of 15 mmHg on both eyes. The fundus examination and posterior segment optical coherence tomography (OCT) were normal.

The tapering of methylprednisolone was initiated, with the continuation of tobramycine-dexamethasone only. During follow-up, the patient developed an increase of IOP, predominantly in the right eye measured at 27 mmHg, attributed to significant pigmentation in the anterior chamber. A treatment with carbonic anhydrase inhibitors and local alpha-adrenergic inhibitors was then initiated.

**Figure 3:**
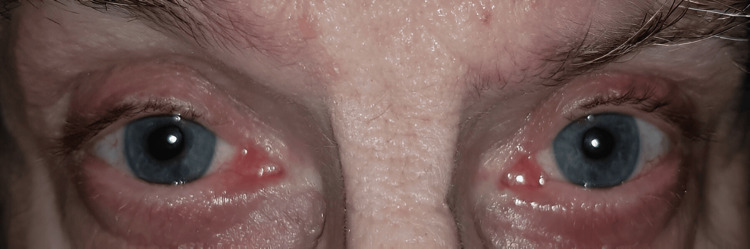
Photograph of the eyes during the one-year follow-up showing persistent synechia and depigmentation zones

**Figure 4:**
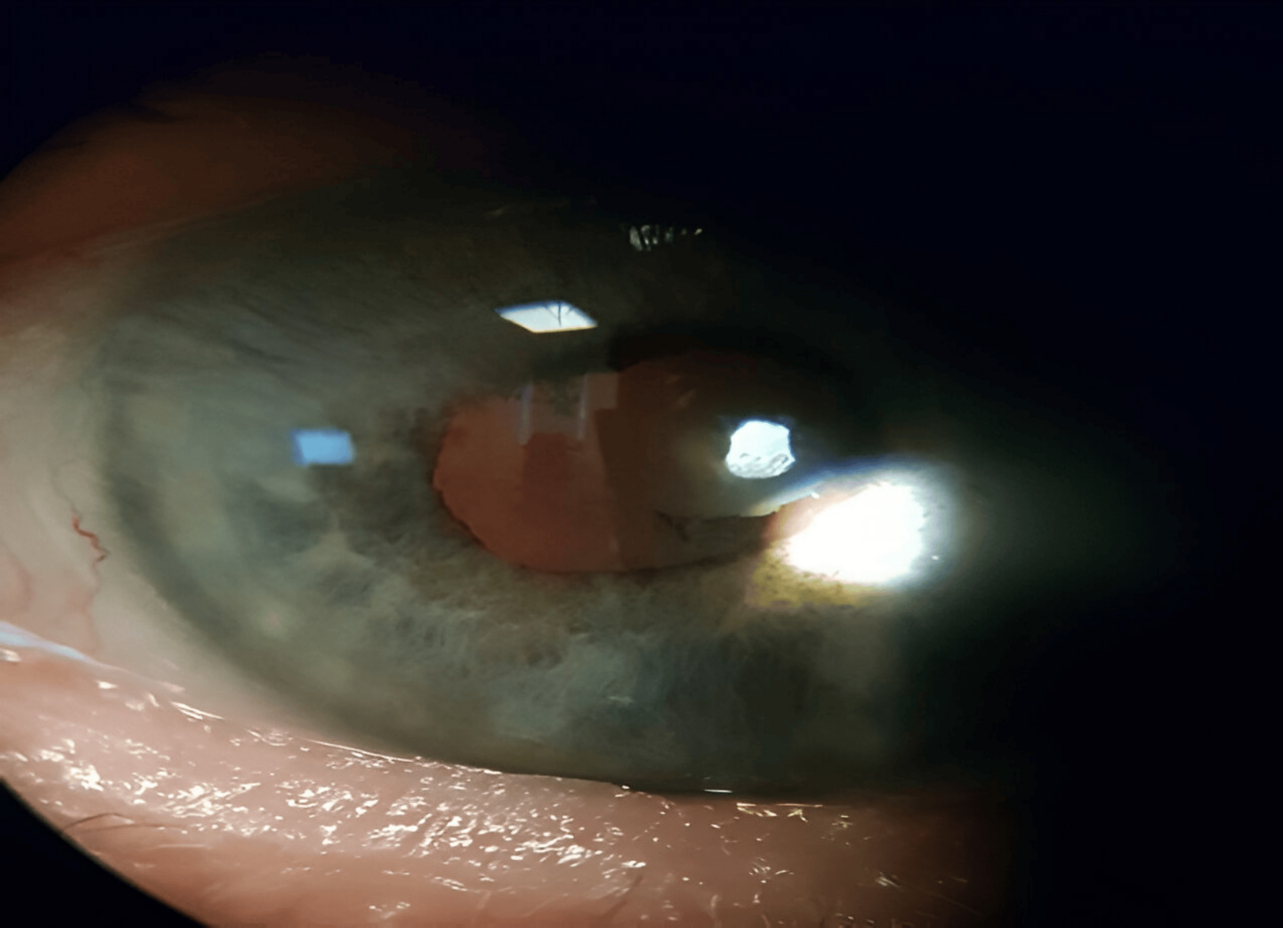
Slit lamp photograph of the left eye showing stromal depigmentation zones from the peripheral iris to the peripupilary area with spared sectors that retain normal iris color

Nearly one year after treatment, the patient showed improved visual acuity, reduced symptoms, and IOP within a normal range. The anterior chamber remained deep, and the angle remained open and free of synechiae, although persistent pigment was noted in the anterior chamber. The lens showed pigment deposits on the anterior capsule, but no anterior subcapsular cataract was present. Nonetheless, persistent iris depigmentation (Figures 3, 4), with large areas of transilluminable diffuse atrophy, was observed.

## Discussion

The presented case illustrates an interesting form of BAIT, where our patient demonstrated key clinical features of BAIT, including diffuse iris transillumination, atonic mydriatic pupils, and persistent pigment dispersion in the anterior chamber, with the additional finding of bilateral bullous keratopathy, demonstrating that severe cases of BAIT can present with significant ocular structural alterations.

Also, the clinical evolution in this case shows the importance of careful monitoring during corticosteroid tapering. The resolution of acute symptoms under systemic corticosteroids was followed by a recurrence of IOP elevation upon dose reduction, reflecting the risk of recurrent symptomatic pigment discharge especially when associated with rapid tapering or early discontinuation of corticosteroids [1]. Such episodes highlight the necessity for regular IOP monitoring and extended follow-up until the resolution of pigment dispersion to prevent long terms sequelae such as glaucomatous damage or irreversible photophobia [2,7]. Pigment circulation in BAIT can persist for months, with a median of five months ranging from one to 18 months [1], compared to BADI, where it generally lasts between one and 16 weeks, with a median of eight weeks [2]. Luckily, in this case, carbonic anhydrase inhibitors and alpha-adrenergic inhibitors were effective in controlling IOP. Nonetheless, some described cases required filtration surgery due to insufficient response to pharmacologic treatment alone [8].

Differential diagnosis was important given the similarities between BAIT with BADI. Both conditions share bilateral pigment dispersion as a hallmark but differ in the origin of pigment loss. In BADI, pigment dispersion occurs from the iris stroma without transillumination defects or atonic pupils, and the condition generally follows a more benign and self-limiting course. By contrast, BAIT involves severe pigment release from the IPE, resulting in diffuse iris transillumination and a higher risk of persistent complications, including significant IOP elevation.

Mixed presentations, where one eye displays features of BADI and the other of BAIT, or cases with overlapping characteristics, have been reported [6,9], further complicating the differentiation between these entities. These findings suggest that BAIT and BADI may represent phenotypic variations of a single pathological entity [2].

The etiopathogenesis of BAIT and BADI remains uncertain, but the association between oral moxifloxacin use and symptom onset in this case supports its role as a potential trigger. Interestingly, the occurrence of unilateral BAIT-like syndromes following intracameral moxifloxacin use provides further evidence supporting its role as a direct toxic trigger [10-13]. Given the growing evidence of this association, clinicians should exercise caution when prescribing fluoroquinolones, especially to patients with a history of BADI or BAIT, as re-exposure could exacerbate pigment dispersion and lead to significant ocular morbidity.

Moreover, another differential diagnosis considered initially was herpetic iridocyclitis, which can mimic BADI and BAIT [2,14]. Herpetic involvement is often unilateral and associated with atrophy of the iris with transillumination defects and elevated IOP [15] as seen in BAIT. Fuchs' iridocyclitis, often associated with heterochromia, represents another differential diagnosis [16]. Similarly, pigment dispersion syndrome (PDS) should also be considered, and is distinguished by the characteristic pigment deposition along Scheie’s stripe on the posterior lens capsule [2]. Finally, in cases of BAIT, it is essential to differentiate it from chronic angle-closure glaucoma (CACG). In CACG, the pupil may be unreactive, remaining mid-dilated and fixed due to ischemia of the iris sphincter, whereas in BAIT, it may be unreactive due to posterior synechiae. BAIT presents with bilateral iris transillumination defects and open angles, whereas CACG is typically characterized by a shallow anterior chamber (usually unilateral) and angle closure.

## Conclusions

This case highlights the clinical variability of BAIT and reinforces the importance of individualized management, particularly during corticosteroid tapering. Accurate differentiation from BADI and other pigment dispersion syndromes is critical to ensure appropriate treatment and follow-up. Additionally, the potential role of moxifloxacin as a trigger underscores the need for vigilance when prescribing fluoroquinolone antibiotics, particularly in at-risk populations. Long-term follow-up remains essential to address potential recurrences and manage chronic sequelae.
